# Creation and validation of an educational video for deaf people about cardiopulmonary resuscitation^*^

**DOI:** 10.1590/1518-8345.2765.3130

**Published:** 2019-03-18

**Authors:** Nelson Miguel Galindo-Neto, Ana Carla Silva Alexandre, Lívia Moreira Barros, Guilherme Guarino de Moura Sá, Khelyane Mesquita de Carvalho, Joselany Áfio Caetano

**Affiliations:** 1 Instituto Federal de Educação, Ciência e Tecnologia de Pernambuco, Campus Pesqueira, Pesqueira, PE, Brazil.; 2 Universidade Estadual Vale do Acaraú, Departamento de Enfermagem, Sobral, CE, Brazil.; 3 Universidade Federal do Piauí, Programa de Pós-Graduação em Enfermagem, Teresina, PI, Brazil.; 4 Universidade Federal do Piauí, Colégio Técnico de Bom Jesus, Bom Jesus, PI, Brazil.; 5 Universidade Federal do Ceará, Programa de Pós-Graduação em Enfermagem, Fortaleza, CE, Brazil.

**Keywords:** Cardiopulmonary Resuscitation, Teaching Materials, Audiovisual Aids, Instructional Films and Videos, Health Education, Persons with Hearing Impairments, Reanimação Cardiopulmonar, Materiais de Ensino, Recursos Audiovisuais, Filmes e Vídeos Educativos, Educação em Saúde, Pessoas com Deficiência Auditiva, Reanimación Cardiopulmonar, Materiales de Enseñanza, Recursos Audiovisuales, Películas y Vídeos Educativos, Educación em Salud, Personas com Deciciencia Auditiva

## Abstract

**Objective:**

to create and validate an educational video for teaching deaf students about cardiopulmonary resuscitation.

**Method:**

methodological study consisting in the creation of an educational video, validation of its content by 22 nurses with expertise in cardiorespiratory arrest, and evaluation by 16 deaf students. For data collection, the following validated instruments were used: the Instrument for Validation of Educational Content used for the validation by nurses and the Assistive Technology Assessment Questionnaire for the evaluation by deaf students. The criterion for validation was concordance higher than 80%, analyzed through the content validation index and binomial test.

**Results:**

the final version of the video lasted seven minutes and thirty seconds, covered the steps that should be performed by a lay person to help a victim of cardiorespiratory arrest, presented animations and the narration in the Brazilian sign language. All the items obtained agreement among nurses and of among deaf students equal or superior to 80%.

**Conclusion:**

the video was considered to present valid content by the judges and comprehensible content by deaf students. Thus the video represents an inclusive technology for health education of deaf people about cardiopulmonary resuscitation.

## RESUMO

**Objetivo:**

construir e validar vídeo educativo para ensino de alunos surdos acerca da ressuscitação cardiopulmonar.

**Método:**

estudo metodológico composto pela construção do vídeo educativo, validação de conteúdo por 22 enfermeiros com *expertise* em parada cardiorrespiratória e avaliação por 16 alunos surdos. Para coleta dos dados, utilizaram-se instrumentos validados: Instrumento de Validação de Conteúdo Educacional para validação pelos enfermeiros e Questionário de Avaliação de Tecnologia Assistiva para avaliação dos alunos surdos. O critério para validação foi concordância superior a 80%, analisada por meio do índice de validação de conteúdo e teste binomial.

**Resultados:**

a versão final do vídeo possuiu 7 minutos e 30 segundos, contemplando as etapas que devem ser realizadas pelo leigo para socorrer a vítima de parada cardiorrespiratória, apresentadas com animação e narração na língua brasileira de sinais. Todos os itens obtiveram concordância dos enfermeiros e dos alunos surdos igual ou superior a 80%.

**Conclusão:**

o vídeo foi considerado válido quanto ao conteúdo, por juízes, e compreensível, por alunos surdos, de forma que consiste em uma tecnologia inclusiva para educação em saúde de surdos acerca da ressuscitação cardiopulmonar.

## RESUMEN

**Objetivo:**

construir y validar un vídeo educativo para enseñanza de alumnos sordos acerca de la reanimación cardiopulmonar.

**Método:**

estudio metodológico compuesto por la construcción de un vídeo educativo; validez de contenido por 22 enfermeros con dominio en paro cardiorrespiratorio y evaluado por 16 alumnos sordos. Para la recolección de los datos fueron utilizados los instrumentos: Instrumento de Validez de Contenido Educacional para validez por los enfermeros y Cuestionario de Evaluación de Tecnología Asistida para evaluación de los alumnos sordos. El criterio para validación fue tener una concordancia superior a 80%, analizada por medio del índice de validez de contenido y prueba binomial.

**Resultados:**

la versión final del vídeo poseía siete minutos y treinta segundos, contempló las etapas que deben ser realizadas por el lego para socorrer la víctima de paro cardiorrespiratorio, presentadas con animación y narración en la lengua brasilera de señas. Todos los ítems obtuvieron concordancia de los enfermeros y de los alumnos sordos igual o superior a 80%.

**Conclusión:**

el vídeo fue considerado válido cuanto al contenido, por jueces, y comprensible, por alumnos sordos, de forma que consiste en una tecnología inclusiva para educación en salud de sordos acerca de la reanimación cardiopulmonar.

## Introduction

Recommendations from the American Heart Association highlight the relevance of teaching cardiopulmonary resuscitation (CPR) to lay people^1^ because of its association with greater survival chance of cardiac arrest (CA) victims in the prehospital setting^2^. Thus, it is important to invest in efforts so that such education can be offered to the maximum number of people who have the capacity to identify a case of CA and physical conditions to perform CPR^3^.

Among the target public are the deaf people, who have no cognitive or motor impairment and only differ from the majority of the population for their auditory impairment. Although physically and mentally fit, they are in disadvantage when it comes to accessing information about identification of a CA and performance CPR, as they face communication barriers and a scarcity of available health education material in sign language^4^ ^-^ ^5^.

Considering that the use of technological resources contributes to the success of health education and that communication with the deaf occurs through visual means, it is worth highlighting the feasibility of using videos as a resource for the provision of health information^6^, according to observed in a study conducted in California whose results showed the effectiveness of a video about cancer aimed at health education for deaf people^7^.

Video also represent an effective option for teaching lay people regarding CPR^8^ ^-^ ^9^. So, considering that videos are an effective resource for teaching deaf people and for multiplying information on CA, it is necessary to build and validate a video about CPR accessible to the understanding of the deaf people.

The construction and validation of an educational video for deaf people about CPR is relevant because it provides a didactic resource that can be used for training of a great number of people with standardized instructions and also corroborates with a self-directed and flexible learning process, because it provides the possibility of autonomy for the learners to watch the video in any moment they prefer and as many times as they deem it necessary^8^ ^-^ ^10^. Such educational technology can also contribute to the practice of health and education professionals who work with deaf students, to enable accessibility and empowerment to act correctly in cases of CA.

Moreover, it is worth noting that nursing has a relevant role in health education, because nurses carry out their professional activities in the various types of health services. They make up the professional category with the greatest number of professionals in the health area, and because they have education as an intrinsic trait of their professional practice. Thus, studies about the construction and validation of educational technologies about CPR are relevant to nursing because they inform about a resource option that can be used in educational interventions.

Thus, this study aimed to create and validate an educational video for teaching deaf students about cardiopulmonary resuscitation.

## Method

Methodological study consisting in the creation of an educational video, validation by judges, and evaluation by deaf students representatives of the target audience.

Recommendations for the construction of audiovisual materials were followed, from pre-production (storyboard planning and design), production and post-production^11^. The initial step was the construction of the storyboard, which consists of the graphic sequence of actions (similar to a comic strip), with a layout that accurately corresponds to what will be the final product^11^.

The selection of the content about safety of the scene, correct identification of CA, call for help, and realization of the CPR followed the recommendations directed to lay people of the Brazilian Society of Cardiology, the American Heart Association, and the Asian and European Councils of Resuscitation^1^ ^,^ ^8^ ^,^ ^12^ ^-^ ^13^.

The storyboard with the scenes and drawings of the animations was built by a design company hired for this purpose under the supervision of the researcher. The choice for the development of video with digital animations instead of scenes recorded with actors was due to the fact that animations allow aesthetic improvement of the drawings, besides requiring less time for production and having a more attractive appearance than the recordings of scenes with actors.

After construction, the storyboard was submitted to content validation by judges with expertise in Basic Life Support (BLS). The sample calculation was performed with the equation for finite populations n = Za^2^.P (1-P)/e^2^. In this equation, Za (confidence level) was stipulated in 95%, P (proportion of experts agreeing with the item) was set at 85% and “e” (expected difference) was 15%, so that the calculated sample was 22 participants.

In order to recruit participants for this stage, the snowball technique was used: electronic contacts of teachers of undergraduate nursing courses and specialization in urgency and emergency and intensive therapy were sought in the websites of the state and federal public institutions located in Fortaleza - CE. These teachers were contacted via e-mail, and they, in turn, indicated other teachers from the states of Pernambuco, Rio de Janeiro, São Paulo and Minas Gerais who had an eligible profile to integrate the sample of this stage of the study.

The inclusion criteria used were: to be a nurse with experience of CPR care and to have teaching or research experience in this theme. The exclusion criterion was incomplete completion of the collection instrument. Thus, 45 professionals were contacted via e-mail (via Google form), in which they received the Informed Consent Term, the storyboard, and the Instrument for Validation of Educational Content (IVEC), which consisted of an instrument with 18 questions (covering the relevance, presentation, structure and objective) built and validated by the research group of assistive technologies for the health promotion of people with disabilities of the Federal University of Ceará. Thus, the 22 professionals who first sent a response were included in the study.

It should be noted that the adjustments made, suggested by the judges, were related to the reformulation of some sentences to improve the chance of the contents to be understood by people without training in the health area. After completing the validation of the storyboard, the video was produced by the same design company that built the storyboard. Three characters were created: a victim affected by CA and a couple of lay rescuers. The images were first drawn in pencil, then vectorized in Corel Draw, converted into a Photoshop object and animated in After Effects.

The content of the video was presented in the order of steps that should be followed to correctly provide help to the CA victim: observation of safety of the scene; identification of the problem; call for help from the Mobile Emergency Service (SAMU); and CPR without interruptions until the arrival of health professionals, with emphasis on the need of relay between rescuers who perform the compressions every two minutes.

Since the objective was that the deaf students understood the content, in the construction of the video the whole narration was made in Brazilian Sign Language (LIBRAS). Thus, the narration was recorded in Libras in a studio by an interpreter of the Institute of Education of Deaf people of Ceará. It should be noted that the text of the narration was on the storyboard that had been previously validated during the stage of validation of content by judges. Synchronization of the components of the video (animation, audio narration and Libras) was performed in the Premiere program, and the interpreter was exposed in the lower right corner, in an area that corresponded to 1/6 of the screen.

As the video is a technology aimed at teaching and demonstrating information, its preparation focused on the reference of Gagné’s instructional events. This reference includes components necessary for the content of an instruction to promote cognitive activation, mental processing, and long-term memory storage. These events focus on attracting the attention of student, informing objectives, stimulating memories and previous knowledge, presenting visual stimuli (images, arrows and flowcharts) and exposing content in organized blocks, with increasing order of complexity^14^.

In the construction of the video, the first event (catching the attention of the student) was contemplated by the presentation, at the beginning of the video, of questions about the need to know how to act in cases of cardiorespiratory arrest. The second event (presentation of the objectives) was shown before the presentation of the objective, with emphasis on the fact that a correct attitude may make a huge difference in saving lives. The third event (stimulus to memories of previous knowledge) was observed in front of the presentation of questions about what the viewer knew about the theme.

**Figure 1:**
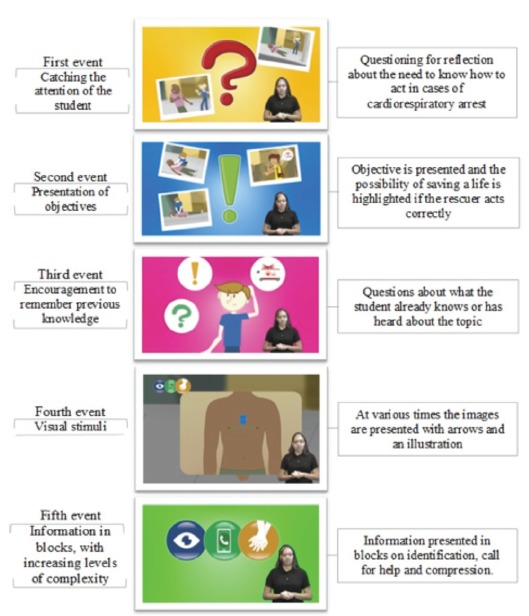
Compliance of the educational video about cardiopulmonary resuscitation for deaf students with the instructional events of Gagné. Fortaleza, CE, Brazil, 2018

The fourth event of Gagné (visual stimuli) was present in the form of schemes/flowcharts and arrows in the illustration of the narrated content. The fifth event, related to the information presented in blocks, was also contemplated, since the content was organized and exposed in three blocks (about the identification of the problem, call for help, and realization of CPR). The video’s compliance with Gagné’s instructional events is summarized in Figure 1.

At the end of the preparation, the video was watched by deaf students from the Institute of Education of Deaf people of Ceará, in the city of Fortaleza/CE, for them to offer opinions about the clarity and understanding of the educational technology. The Institute of Education of Deaf people of Ceará, which is the only public institution exclusive dedicated for the education of deaf students in Ceará, has primary and secondary school classes and had, during the data collection period, approximately 70 students enrolled in each shift (morning, afternoon and evening), totaling about 210 students.

The inclusion criteria for selection of deaf students to evaluate the material were: to be enrolled in the educational institution and not to have cognitive limitations incompatible with the evaluation of the video. It should be noted that it was possible to check the existence of cognitive limitation based on information provided by the schools, because they were adapted to the teaching of deaf students and had, in the registers of enrollment, medical evaluation reports with details and attestation of deafness, pathologies, as well as motor and cognitive limitations. These records were consulted to confirm the absence of cognitive limitations in the students who participated in this study. The incomplete completion of the instrument was the exclusion criterion used.

Recruitment occurred at the break between classes, in a day, shift and schedule defined by the head office of the institution. At that moment, students were approached in the courtyard and, with the help of a Libras interpreter, they were invited to go to the audiovisual room of the school reserved for evaluation. In view of this recruitment strategy, there was no calculation to define the number of participants that would be part of the sample of this stage of the study; this number was defined by the number of students who agreed and volunteered to participate. Thus, 16 students agreed to participate in the study and signed the ICF. The video was presented on a 32-inch television, with deaf students accommodated in school desks.

After watching the video, the participants completed the Assistive Technology Assessment Questionnaire^15^, which is a validated instrument for verifying the understanding of assistive educational technologies. The instrument has 14 questions that address the objectives (if the technology encourages learning, if the acquisition of new concepts facilitates the search for information, and if it is attractive), clarity (if the content of the technology is presented in a simple manner and stimulates reflection); relevance (if the technology presents resources to make its use viable, if it stimulates the interest in its use, if it encourages the adoption of new behaviors, and if its content can be applied in different contexts); and interactivity (if the material is suitable to be used, if it allows interaction, if it enables autonomy, and if is accessible without difficulties).

The researcher read the instrument, which was simultaneously translated by the Libras interpreter. One question at a time was read and translated; after the reading (of the researcher) and the translation into Libras (of the interpreter) of each question, it was expected that all the deaf students answered of the question explained, so that the next question was read and interpreted. Thus, all deaf students simultaneously filled out each of the questions on the instrument.

The analysis of the data was carried out in the R software, version 3.1.1. In order to consider the validation, the Content Validation Index (CVI) was used in three ways: judges’ agreement on each item with the I - CVI (Item-level Content Validity Index); proportion of items in which there was agreement of each judge, with the S-CVI/AVE (Scale-level Content Validity Index, Average Calculation Method); and the mean S-CVI/AVE, which is the S-CVI (Scale-level Content Validity Index). The binomial test, with significance level of 5%, was used to verify if the agreement ratio was statistically equal to or greater than 0.8^16^.

The research was developed as established by Resolution 466/12 and was approved by the Ethics Committee of the Federal University of Ceará (Opinion 2,108,475).

## Results

**Figure 2:**
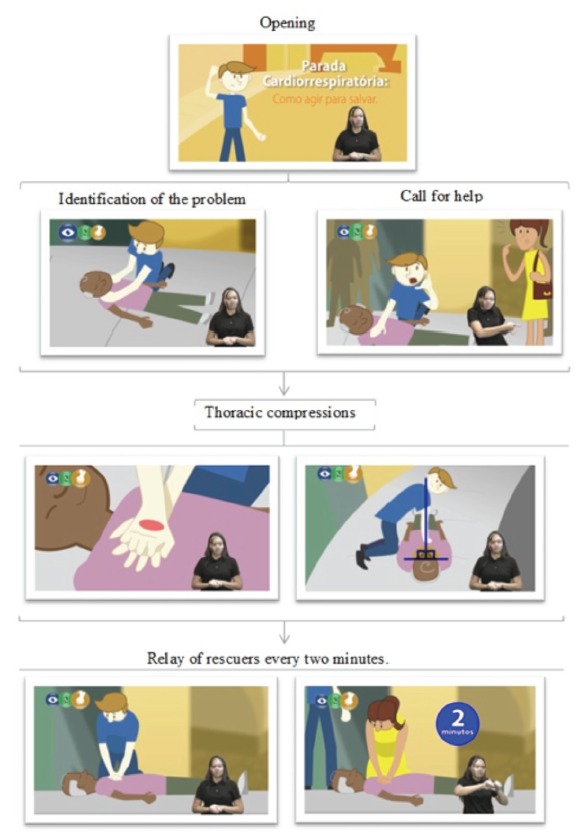
Sequence of information in the content of the educational video for deaf people about cardiopulmonary resuscitation. Fortaleza, CE, Brazil, 2018

The final version of the video had a duration of 7 minutes and 30 seconds and was composed by the opening, questions about the need to know how to act correctly in cases of cardiorespiratory arrest, presentation of the objective, questions regarding the prior knowledge of the spectator, information about the importance of safety in the scene, and three blocks of content, namely: information regarding the correct way to identify the problem; calling for help, highlighting the correct number for calling the SAMU; and performance of CPR, as observed in Figure 2.

Thus, the educational video (available for access at: https://www.youtube.com/watch?v=V6_CnIn6TOo&t=1s) has accessibility in Libras to contribute, as a technological resource, to the teaching-learning of deaf students about the correct provision of assistance to victims of CA in an out-of-hospital setting.

The judges who validated the content were all graduates of nursing course, four (18.2%) had a master’s degree, five (22.7%) had PhD degree, and the others were specialists. Regarding the professional activity, all had previous experience in urgency and emergency care or intensive care, and at the time of data collection, 15 (68.2%) were teaching in higher education institutions and/or specialization courses, and they provided content related to CPR. All had already participated in courses on CPR and 17 (77.3%) had already taught these types of courses. Moreover, 18 (81.8%) had published scientific articles on CPR in periodicals or in annals of events.

In the validation of storyboard content presented in the video, there was a minimum 86% of agreement on the objectives, structure and presentation. Among the items referring to relevance, the lowest agreement was 77% in the item about adequacy of the size of the material. The five judges who disagreed with this item reported that the time programmed for the video (approximately seven minutes) was too long and did not present suggestions of adjustments/modifications, however, as the binomial test of such item was not significant, such agreement was considered statistically equal to or greater than 80%, so that the item was considered valid and there was no change.

There was 86% agreement of the judges in the item “the information has accurate content”. The three judges who disagreed presented as justification the need of the educational material to show/teach how to check the carotid pulse in the identification of CA. It is worth noting that it was impossible to meet this suggestion because the protocols of the American Heart Association recommend that the presence of pulse is an action indicated for health professionals (due to the need for minimal knowledge on anatomical traits) and that lay rescuers should detect a CA if the individual is unconscious and in apnea.

Furthermore, there was unanimity of agreement among the judges in the items concerning the fact that the video favors a reflection on the theme, deals with a modern theme, and collaborates with the area of knowledge (Table 1).

**Table 1:** Judges’ agreement on the objectives, structure, presentation and relevance of the educational video. Fortaleza, CE, Brazil, 2018

**Items**	**Judges’ Agreement**		**p** ^**†**^
	**n (%)**	I-CVI*	
**Objectives**			
**1. Contemplates the theme**	**21 (95.5)**	**0.95**	**0.972**
**2. Is suitable for teaching-learning**	**20 (90.9)**	**0.90**	**0.863**
**3. Enables the clarification of doubts**	**19 (86.4)**	**0.86**	**0.661**
**4. Favors reflection on the theme**	**22 (100.0)**	**1**	**1**
**5. Influences the adoption of new behaviors**	**20 (90.9)**	**0.90**	**0.863**
**Structure and presentation**			
**6. Language compatible with the understanding of the public**	**20 (90.9)**	**0.90**	**0.863**
**7. Language suitable for the type of material**	**21 (95.5)**	**0.95**	**0.972**
**8. Language prompts interaction/involvement**	**20 (90.9)**	**0.90**	**0.863**
**9. Information with accurate content**	**19 (86.4)**	**0.86**	**0.661**
**10. Objective presentation of content**	**20 (90.9)**	**0.90**	**0.863**
**11. Clear presentation of content**	**20 (90.9)**	**0.90**	**0.863**
**12. The content shown is indispensable**	**19 (86.4)**	**0.86**	**0.661**
**13. Ideas exposed in a logical way**	**20 (90.9)**	**0.90**	**0.863**
**14. The theme is modern**	**22 (100.0)**	**1**	**1**
**15. The size is adequate**	**17 (77.3)**	**0.77**	**0.226**
**Relevance**			
**16. Encourages learning**	**21 (95.5)**	**0.96**	**0.972**
**17. Collaborates with the knowledge area**	**22 (100.0)**	**1**	**1**
**18. Stimulates interest in the theme**	**21 (95.5)**	**0.95**	**0.972**

*Item-level Content Validity Index; ^†^Binomial test

Regarding the S-CVI/AVE of the content validation, 12 judges disagreed with two items (agreement with 88.8% of the items and S-CVI/AVE = 0.88); six judges disagreed with one item (agreement of 94.4% and S-CVI/AVE = 0.94); and four judges agreed with all items (S-CVI-AVE = 1). Thus, the minimum S-CVI/AVE was 0.88 and the S-CVI of the content validation was 0.91.

Among the deaf students who evaluated the video, 11 (68.7%) were women, 14 (87.5%) were single, none had children and all were in high school.

According to the evaluation of these students, the video was considered comprehensible and there was a minimum agreement of 87% in the item “the acquisition of new concepts facilitates the search for information”. The deaf students who disagreed with this item argued that the search for information would only be facilitated if the viewer had control over the video projection technology so that they could pause and review some parts where necessary.

In addition, there was a minimum agreement of 93% regarding the relevance and efficacy and unanimous agreement on clarity and interactivity, as observed in Table 2.

**Table 2:** Deaf students’ agreement on interactivity, objectives, relevance, effectiveness and clarity of the educational video. Fortaleza, CE, Brazil, 2018

**Questions**	**Deaf students’ agreement**		**p** ^**†**^
	**n (%)**	I-CVI*	
**Interactivity**			
**1. Material with suitability for its use**	**16 (100.0)**	**1**	**1**
**2. Material enables interaction**	**16 (100.0)**	**1**	**1**
**3. Access is easy**	**16 (100.0)**	**1**	**1**
**4. It enables autonomy for its use**	**15 (93.7)**	**0.93**	**0.937**
**Objectives**			
**5. Encourages learning**	**16 (100.0)**	**1**	**1**
**6. Encourages the acquisition of new concepts**	**16 (100.0)**	**1**	**1**
**7. Facilitates the search for information**	**14 (87.5)**	**0.87**	**0.875**
**8. Has an attractive presentation**	**16 (100.0)**	**1**	**1**
**Relevance and effectiveness**			
**9. Presents resources to enable its use**	**16 (100.0)**	**1**	**1**
**10. Stimulates the interest in its use**	**15 (93.7)**	**0.93**	**0.937**
**11. Encourages the adoption of new behaviors**	**16 (100.0)**	**1**	**1**
**12. The content can be applied in various contexts**	**16 (100.0)**	**1**	**1**
**Clarity**			
**13. Content is presented in a clear way**	**16 (100.0)**	**1**	**1**
**14. Stimulates reflection**	**16 (100.0)**	**1**	**1**

*Item-level Content Validity Index; ^†^Binomial Test

Regarding the S-CVI/AVE of the evaluation by the deaf students, four disagreed with one of the 14 items, so that there was agreement in 93.7% of the items and the S-CVI/AVE of each one was 0.93. The other 12 deaf students agreed on all items (S-CVI/AVE = 1). Thus, the S-CVI of the evaluation of deaf students was 0.98.

## Discussion

The content that subsidized the construction of the video contemplated the safety of the scene, correct identification of the problem, call for help, and correct way to perform the CPR. These contents were relevant to be addressed in the video, because, once performed correctly, contribute to reduce the probability of death.

Regarding the first information presented by the video (safety of the scene), the American Heart Association and the European Resuscitation Society emphasize the need to verify such safety before any approach to the victim^1^ ^,^ ^9^ ^,^ ^17^. Results from Norwegian research with lay people who rescued patients who had undergone a CA in out-of-hospital settings showed that the situation of rescuing is permeated by worry and nervousness^18^. It is, therefore, understandable that the attention given to the victim may lead to negligence about the safety of the place, so that the risks that may have affected the victim can also cause injury to the rescuer. Thus, attention to the safety of the scene in the video is important to contribute to the dissemination of this necessary precaution.

Other relevant steps presented in the sequence of the content of the video concern the correct way to identify a CA and the request for help. When considering the incompatibility of life with the CA, fast assistance of specialized professionals becomes of major importance. However, the access of health professionals to the victim depends directly on the identification of the grievance, so that the mobile medical service be called right away^8^.

As soon as an out-of-hospital CA is identified, the task to call the mobile emergency service^12^ ^-^ ^13^ must be assigned to someone. However, what happens in real cases is that people not only wait and trust that someone will have the idea to call the health professionals, but also are still unaware of the correct telephone number of the emergency service, according to a research in the state of Minas Gerais showed. In this study, 47.3% of the 401 study participants were unaware of the correct number of Emergency Mobile Service (SAMU)^19^. It is, therefore, important to highlight the relevance of the educational video to present information about the telephone contact of the Brazilian prehospital service.

The content used in the construction of the educational video also included, in particular, the correct realization of CPR, which explains the position of the rescuer, the point of the victim’s chest where the compressions must applied, the rate and depth that characterize high quality compressions^12^ ^-^ ^13^.

The correct realization of these conducts is related to the effectiveness of the CPR and the probability of return of spontaneous circulation, as it is pointed out in the results of a North American multicenter study carried out with 8719 participants, where there was a correlation of correct rate and depth with the lowest mortality^20^. When considering that the correct performance of CPR is made possible by the correct position of the rescuer and the correct positioning of his hands upon the middle of the victim’s chest, it is pertinent to point out that the detailed presentation of these items in the educational video may contribute to a greater probability of correctly realization of CPR, and with higher quality.

Despite the rigorous construction based on scientific literature on the subject, the validation of the content of the video was important as well as its evaluation by deaf students, for scientific anchoring and attributing credibility to this educational technology.

Regarding the identification of CA, some judges disagreed with the content because they thought the video needed to inform the need to check the carotid pulse to identify a CA. However, as individuals who do not respond and do not breathe have a high chance of being in CA, and because confirming the presence of a palpable central pulse is pertinent to health professionals, it is recommended that the population be oriented to treat as a CA all cases where there is no responsiveness or breathing^3^ ^,^ ^12^ ^-^ ^13^. Thus, the presence of such content in the video is relevant to multiply information on the identification of CA cases, which will trigger the realization of the subsequent steps of care.

In the view of the nurses who participated in the validation, the video presented sufficient and necessary information. This result corroborates Brazilian methodological researches on the validation of a booklet on first aid at school and a video for parents of children using clean intermittent catheters, whose results showed that the evaluators agreed with the pertinence and adequacy of the content presented^21^ ^-^ ^22^.

These Brazilian findings corroborate a study carried out in Venezuela regarding the construction and validation of an assistive technology for deaf people on oral health, which showed similar agreement among the evaluators^23^. The aforementioned results point to the relevance of the evaluation by specialists about the quality and sufficiency of the content of health education technologies, taking into account that educational materials may neglect important information or present it in a brief, superficial or without the importance that it actually has, which may compromise the effectiveness of the technology.

In the validation of the content, there was agreement that the video had clear information, with a language compatible with the understanding of the public, corroborating the evaluation of the deaf students, in which there was agreement on the comprehension and attractiveness of the video. A Brazilian study of validation of a printed material on prevention of metabolic syndrome, and a research conducted in New York about a video animation aimed at the teaching about the human genome found similar agreement regarding the understanding of the technologies^24^ ^-^ ^25^.

As the health area uses specific terms, it is necessary to evaluate the language used in teaching materials for the population so that the educational technologies do not become of little usefulness and human inputs and materials do not be wasted in the dissemination and distribution of unclear or confusing materials. The incompatibility about the clarity of educational technologies in health is presented in a study whose results showed that 80% of the virtual contents of the American Academy of Orthopedic Surgery used in patient health education were incomprehensible to the target audience. Similar findings were presented in a study in Germany, whose results showed that educational materials on ophthalmology from 32 hospitals were not understood by the population for which they were created^27^.

In this context, it is necessary that educational technologies be evaluated by representatives of the public who will use them, so that confusing and little comprehensible parts are adjusted and become compatible with popular understanding. Thus, the agreement of nurses and deaf students regarding the clarity of the video on CPR corroborates with the greater probability of the technology to enable the multiplication of information and contribute more effectively with the training of lay people on the subject.

According to the evaluation of deaf students, the educational video stimulates interest and learning. Similar findings were observed in a research with schoolchildren in Indonesia on a video animation about metabolism^28^. Given the relevance of motivation and popular adhesion, in order to make viable the use of educational technological resources, it is pertinent to highlight the need to consult such the target population of the technologies so that requests for adjustments may be considered in order to contribute to the improvement of the material and the greater probability of success of its use.

The positive evaluation of deaf students is relevant because, although the video was considered long by five judges (who disagreed about the adequacy of its size), the agreement of deaf students about the clarity of the content and the stimulation to learn confirmed that the duration of the video was necessary to properly expose the content, in a manner compatible with the understanding.

The longer of time needed in the exposition of the information is due to the synchrony of the video directed to deaf students needs to respect the speed of capture of visual stimuli of this public, because the deaf spectator needs first observe the narration in Libras for then leave the visual field of the Libras interpreter to observe the demonstrations and animations. Thus, scenes and demonstrations need to be presented/displayed slower to allow the deaf student to see both the narration in Libras and the demonstrations/animations. This aspect makes sign language videos longer than videos with audio narration only.

The educational video on CPR was considered adequate and easy to use by the deaf students who evaluated it. Similar results were found in a study that evaluated electronic technology for teaching sign language in Bogotá and in a Brazilian study that included assistive technology for the blind people about breastfeeding^29^ ^-^ ^30^. Although the deaf students considered it easy to use the video, some of them disagreed about whether it was easy to seek information in the video, because they cannot control the technological resources that allowed them to pause and/or review some parts of the material.

In this context, it is necessary to stress the need to empower the population to use educational technologies, especially deaf students, in order to stimulate autonomy, so that the search for information is facilitated. The findings also point to the relevance of investigating the opinion of the target population about the use of technologies so that the final version available is as compatible as possible with their autonomy. This corroborates the active participation of the population in the processes of teaching-learning about prevention, recovery and rehabilitation in health.

The agreement among deaf students was influenced not only by the narration of the video in Libras, but also by the use of the Gagné’s theoretical reference in its construction. It is worth noting that the events related to the presentation of the content in blocks (of increasing complexity) and to the exhibition of visual stimuli (arrows, diagrams, images) converge to benefit the understanding of the deaf students, due to the exacerbation of their visual perception. Thus, in methodological researches, it is pointed out the necessity of using references that fit and contemplate specific aspects of the target audience to which the technologies are intended.

As a limitation out of the study is that the research was conducted in one Brazilian state and the participants were students. It is possible that its findings are distinct from the other states or from the reality of deaf people who are not students.

It is worth noting that the construction and validation of the educational video about CPR adapted for use with deaf students contribute to the advancement of scientific knowledge in view of the availability of a didactic resource constructed with basis on an educational reference (Gagné’s instructional events) and technical and scientific rigor, attesting to the validity of the content of the material as well as its comprehension by deaf students. The video can be used in the teaching-research-outreach activities tripod and enable the access to information about the CPR for deaf students.

Considering that the role of health educators is inherent to the professional practice of nursing and that this category works in the assistance to CA and therefore has the expertise to carry out educational interventions about CPR, the construction and validation of the video about this topic is very relevant. Furthermore, this methodological study provides a replicable method to be used in nursing science for the construction and validation of other assistive technologies, with accessibility in Libras, in future studies.

It is also worth noting that the availability of the educational video in Libras, with valid content and comprehensible for deaf students, not only makes it possible to use them during educational interventions, but also for self-instruction to build knowledge even in the absence of health professionals/instructors. In addition, the material contributes to the multiplication of information about CA, a theme little disseminated in Libras, for a public who, once trained to act correctly, may intervene and contribute to reducing mortality.

## Conclusion

An educational video on cardiopulmonary resuscitation was developed for deaf students. Its final version lasted 7 minutes and 30 seconds. It dealt with the sequence of behaviors to be adopted by a lay person for the correct provision of help for a victim of CA, and had its narration presented in audio and in Libras, in which the interpreter occupied about 1/6 of the screen.

The validation of content had over 80% of agreement in all items, regarding objectives, presentation and relevance, and the S-CVI was 0.91. In the evaluation by deaf students, an agreement greater than 80% was also observed, regarding interactivity, objectives, relevance, effectiveness and clarity, and the SCV-I was 0.98. By means of the binomial and CVI tests, in the forms of I-CVI, S-CVI/AVE and S-CVI, the validity of the video regarding the content and appearance and its approval by the deaf students was demonstrated.

The video can enable deaf students to access content on CPR and it is, therefore, a viable technological resource to be used by nursing and other health professionals in health education. It is necessary to investigate the effectiveness of using the video in the knowledge and ability of deaf students about CPR.
